# Can upfront DPYD extended variant testing reduce toxicity and associated hospital costs of fluoropyrimidine chemotherapy? A propensity score matched analysis of 2022 UK patients

**DOI:** 10.1186/s12885-022-09576-3

**Published:** 2022-04-26

**Authors:** Apostolos Tsiachristas, Grant Vallance, Rositsa Koleva-Kolarova, Harriet Taylor, Luke Solomons, Giovanni Rizzo, Catherine Chaytor, Junel Miah, Sarah Wordsworth, A. Bassim Hassan

**Affiliations:** 1 Nuffield Department of Population Health, University of Oxford, Richard Doll Building, Old Road Campus, Oxford, OX3 7LF UK; 2 Oxford University Hospitals NHS Trust, Oxford, UK; 3 Sir William Dunn School of Pathology, University of Oxford, Oxford, UK

## Abstract

**Aim:**

To independently assess the impact of mandatory testing using an extended DPYD variant panel (ToxNav®) and consequent dose adjustment of Capecitabine/5-FU on recorded quantitative toxicity, symptoms of depression, and hospital costs.

**Methods:**

We used propensity score matching (PSM) to match 466 patients tested with ToxNav® with 1556 patients from a historical cohort, and performed regression analysis to estimate the impact of ToxNav®on toxicity, depression, and hospital costs.

**Results:**

ToxNav® appeared to reduce the likelihood of experiencing moderate (OR: 0.59; 95%CI: 0.45–0.77) and severe anaemia (OR: 0.55; 95%CI: 0.33–0.90), and experience of pain for more than 4 days a week (OR: 0.50; 95%CI: 0.30–0.83), while it increased the likelihood of mild neutropenia (OR: 1.73; 95%CI: 1.27–2.35). It also reduced the cost of chemotherapy by 12% (95%CI: 3–31) or £9765, the cost of non-elective hospitalisation by 23% (95%CI: 8–36) or £2331, and the cost of critical care by 21% (95%CI: 2–36) or £1219 per patient. For the *DPYD* variant associated with critical risk of toxicity (rs3918290), the improved non-elective hospital costs were > £20,000, whereas variants associated with hand-foot syndrome toxicity had no detectable cost improvement.

**Conclusion:**

Upfront testing of DPYD variants appears to reduce the toxicity burden of Capecitabine and 5-FU in cancer patients and can lead to substantial hospital cost savings, only if the dose management of the drugs in response to variants detected is standardised and regulated.

**Supplementary Information:**

The online version contains supplementary material available at 10.1186/s12885-022-09576-3.

## Introduction

In the era of personalised medicines, there are an increasing number of opportunities for clinicians to provide treatments for their patients where drug side effects can be minimised or avoided by upfront testing protocols, often using genetics based tests. In the context of cancer, fluoropyrimidine-based chemotherapy drugs, including Capecitabine and 5-fluorouracil (5FU), have been used widely for decades to treat several solid tumour types in either the adjuvant or palliative setting.^1 2^ Whilst these treatments are usually well received by most patients, with 10–20% experiencing mild toxicity, around 1–5% of patients experience severe or life-threatening toxicity due to significantly impaired function of an enzyme, dihydropyrimidine dehydrogenase (DPYD), that normally acts to catabolise 5-FU [1–3]. Rare variant alleles in the *DPYD* gene have been identified in humans that impair enzymatic activity, with low enzyme activity correlating with the worst degree of side effects following standard-of-care dosing, including mucositis, diarrhoea, hand-foot syndrome (HFS), skin toxicity, tiredness, myelosuppression, and multi-organ failure. This association supports the premise that loss-of-function variants in *DPYD* and other components of the 5-FU catabolic pathway are associated with higher drug levels and increased risk of toxicity.

The Clinical Pharmacogenetics Implementation Consortium (CPIC) 2017 guidelines only support the clinical use of four DPYD variants from many that have been detected in pre-treatment testing, followed by a standardised dose adjustment [4]. Several European countries (e.g. The Netherlands, France, and Italy) have already issued guidelines for the use of the four variant testing and it has been recently recommended for commissioning by NHS England [5, 6]. This is because, besides the clinical significance, there is increasing evidence that upfront genotyping of DPYD in cancer patients assigned to fluoropyrimidine-based chemotherapy may be associated with cost savings and improved quality of life [7–13]. However, the available economic evidence remains limited with respect to mandatory testing despite the recent recommendation of The European Medicines Agency for upfront testing [14]. As a result, the upfront screening for DPYD variants is only just being universally implemented in daily clinical practice (with the exception of the Netherlands) and concerns about its cost-effectiveness have been cited amongst the main reasons for the slow adoption.^9 10 12^

Furthermore, the four CPIC variants have been validated in mainly Caucasian populations based on meaningful allele frequencies that the impact on DPYD detection. However, there is evidence that DPYD variants allele frequencies vary, with some more prevalent in populations of African descent [15]. Moreover, approximately 7–10% of European populations carry at least one *DPYD* variant allele, and in some rare cases both alleles are affected. Despite the use of the four CPIC variant alleles, the risk of toxicity is approximately 4-fold with the severe risk alleles, and 1–3 fold with the high risk alleles, meaning that not all patients experience toxicity despite carrying a risk allele. Importantly, at least 40–50% of patients continue to have severe toxicity after 5-FU or Capecitabine even if they test negative for the four CPIC alleles. This suggests that there remain uncharacterised and rare variants that are also pathogenic; for example, variants associated with severe hand-foot syndrome and variants potentially in other genes associated with fluorpyrimidine induced cardiac toxicity. In support of the need for further pharmacogenomic discovery, several mutant DPYD proteins have been purified and tested in enzymatic assays, and suggest that many more functional variants may exist and need to be considered [11].

In response to the need to consider further variants, an extended ToxNav® panel has been developed based on randomised clinical trial genotyping data by Oxford Cancer Biomarkers in the UK [11].. The panel includes three of the four CPIC alleles (excluding rs56038477/rs75017182 HAPB3) with an additional 15 variants associated with *DPYD* function and one allele of the *ENOSF1* gene. The expectation of this extended panel test, was that variants detected in the clinical trial were more representative of the variants that are functionally important in the real world, and that strong correlation with the toxicity detected in the trial was driving the selection. In ToxNav®, these include an extended number of variants associated with hand-foot syndrome fluorpyrimidine induced toxicity. Despite the anticipated health benefits arising from the ToxNav® panel, it remains unclear whether these benefits would actually be realised in practice, and if there were benefits, whether the panel was likely to be affordable for health care providers. The aim of this study was to independently assess from the manufacturer providing the test, the real-world impact of mandatory testing with ToxNav® and consequent dose adjustment of Capecitabine and 5-FU on recorded quantitative toxicity, perceived patient reported mental health depression scores, and hospital costs per patient.

## Material and methods

### Study design and setting

Our observational retrospective longitudinal study included all cancer patients in Oxford University Hospitals (OUH) NHS Trust who had received either capacitabine or 5-FU chemotherapy, either alone or in combination with other agents, between 1 June 2017 and 1 September 2020. From June 2019, patients who were candidates for 5-FU/Capecitabine therapy routinely underwent mandatory ToxNav® testing prior to treatment initiation. This cohort of patients (i.e. ToxNav) was compared to the historical cohort of patients from 1st June to 31st May 2019 who had received these chemotherapy drugs without ToxNav testing in the same centre (hereafter No-ToxNav).

### Data

We linked routinely collected data from different hospital databases to obtain anonymised individual patient level data for patient demographics (i.e. age, gender and self-reported ethnicity), diagnosis (i.e. ICD10 codes), tumour characteristics, treatment (i.e. regimen, cycles, dose of each drug), adverse events, categorised according to their severity using the Common Terminology Criteria for Adverse Events (CTCAE v6.0) grading system for low count of haemoglobin, neutrophils, and white cells as well as temperature. Using the chemotherapy dose data and date of administration, we defined a variable for initial dose reduction if patients received less than 80% of the dose that should be given based on the treatment protocol at the first cycle of chemotherapy. In addition, we extracted all hospital activity and costs from the OUH’s financial information system for all patients in our study. This data included presentations to accident and emergency (A&E) departments, elective and non-elective admissions to hospital wards, admissions to critical care unit, admissions to day care units, physical or virtual contacts with outpatient clinics, chemotherapy/pharmacological treatment, radiotherapy, diagnostics tests (e.g. lab and imaging test requests), equipment (e.g. wheelchairs, prosthetics, and devices), and rehabilitation (e.g. community reablement team). The dates of each used hospital resource and the respective healthcare resource group (HRG) code and actual price charged by the hospital to the commissioners. The costs of ToxNav® test are not included in the hospital’s financial records and therefore they are not part of our analysis. Costs were inflated to 2020/21 values using the NHS Cost Inflation Index [16]. Data from a routinely administered survey to cancer patients based on the eight-item Patient Health Questionnaire depression scale (PHQ-8) were also linked to the dataset [17]. Finally, we also obtained the ToxNav variant test results of all patients from 1st of June to September 2020 as provided by the test manufacturer. An overview of the regimen included in our sample is provided in Appendix A.

### ToxNav test

In this study, we used the extended ToxNav® (Oxford Cancer Biomarkers) panel that included three of the four CPIC alleles as the PRECISE study had shown that the rs56038477/rs75017182 HAPB3 allele indicated 75% enzyme activity and normal dose of 5-FU/Capecitabine was recomended [18] (more details are provided in Appendix B). In addition, ToxNav® included 15 more variants associated with *DPYD* function and one allele of the *ENOSF1* gene [11]. The test results were categorised by the manufacturer as “Standard” if there was no variant found, “Hand and Foot Syndrome (HFS)” if variants related to HFS were found, and “High” for heterozygous and homozygous variants related to high toxicity (50–75% DPYD activity), and “Critical” for variants associated with little or no DPYD activity (0–50% DPYD activity). The ToxNav® manufacturer recommends no treatment with Capecitabine or 5FU for “Critical” homozygous variants, a 50% dose of these drugs if a patient is tested with either heterozygous “Critical” risk variant or “High” risk of toxicity, and 100% dose in case of Standard or HFS test result, the latter with caution with respect to skin toxicity (see Appendix B).

### Propensity score matching and statistical analysis

As this was an observational study, we followed the Medical Research Council (MRC) guidelines on how to infer causality (i.e. attribute effect to ToxNav in our case) in non-randomised evaluation studies and used the scientific literature to reduce observed confounding between the two cohorts by performing propensity score matching (PSM) [19–23]. We matched the two cohorts by socio-demographic characteristics (i.e. age, gender, ethnicity), diagnosis code (i.e. primary ICD-10 code), treatment (i.e. regimen and number of chemotherapy cycles), duration of follow-up (i.e. the interval between June 2017 and either September 2020 or the date of death), and estimated survival based on all other observed confounders. The latter was included in the PSM as a proxy of cancer severity. We used the whole sample in the PSM and the ratio of cases to controls was approximately 1 to 3. Following best practice, we performed regression analysis using generalised linear regression models (GLMs) in combination with PSM to reduce as much as possible the differences in the confounding variables listed above [24]. A detailed description of the methods used in PSM and regression analysis are provided in Appendix C.

## Results

### Characteristics of population cohorts

Table 1 presents the patient demographics and clinical characteristics for the two cohorts. The ToxNav and No-ToxNav cohorts were similar in terms of age, race, and tumour site (the statistically significant difference in tumour site is due to the higher missing observations in the ToxNav cohort). The ToxNav cohort included less females compared to the No-ToxNav cohort. The fact that the No-ToxNav cohort was a historical control, is reflected to the 11 months longer follow-up period, on average 1.3 more chemotherapy cycles per patient, and the 12 p.p. higher rate of mortality during the observation period. The difference in follow-up time was apparent in the higher mortality rate in the No-ToxNav cohort (32% vs 20%) but the chemotherapy attributed mortality was very similar between the two cohorts.

**Table 1:** Patient demographic and clinical characteristics

	ToxNav (*n =* 466)	No-ToxNav (*n =* 1556)	Difference (*p-*value)
Female	218 (47%)	900 (58%)	−11 p.p. (0.000)
Race			(0.136)
Caucasian	304 (65%)	1079 (69%)	−4 p.p
Asian	6 (1%)	28 (2%)	−1 p.p.
African	2 (0%)	15 (1%)	−1 p.p.
Mixed	0 (0%)	3 (0%)	0 p.p.
Other/unknown	154 (33%)	432 (28%)	5 p.p.
Tumour site			(0.000)
Upper GI	71 (15%)	299 (19%)	−4 p.p.
Lower GI	167 (36%)	724 (47%)	−11 p.p.
Breast	117 (25%)	382 (25%)	0 p.p.
Other	35 (8%)	132 (8%)	0 p.p.
Missing	76 (16%)	19 (1%)	15 p.p.
Mean age at start FU	61.3 (12.9)	60.1 (12.9)	−1 (0.0643)
Mean FU months (SD) n	13.8 (6.2) 391	24.6 (13.0) 1470	−11 (0.0000)
Mean chemo cycles (SD) n	3.8 (2.9)	5.1 (7.8)	1.3 (0.0401)
Total deaths	95 (20%)	490 (32%)	−12 p.p. (0.000)
Deaths within 30 days of chemo	7 (2%)	35 (2%)	0 p.p. (0.321)
Deaths due to Capecitabine/5FU	1 (0.2%)	10 (0.6%)	0 p.p
Cardiac deaths due to Capecitabine/5FU	1 (0.2%)	2 (0.1%)	0 p.p

*P.p* Percentage points, *FU* Follow-up, *SD* Standard deviation, *GI* Gastro-intestinal, Note: race was defined using self-reported ethnicity.

### Propensity score matching

Kernel matching was the PSM technique that achieved the best covariate balance among other tested PSM techniques. The Rubin’s B was 24.3 and R was 0.94 indicating that the achieved covariate balance was within acceptable levels. See Appendix D for more details.

### ToxNav® results and their impact on clinical decision

From the 466 patients tested with ToxNav® variant panel test, 311 (67%) were classified as HFS, 139 (30%) as Standard risk, and 16 (3%) as High and Critical risk (Table 2). This table presents the ToxNav results across patient demographics and the percentage of initial (i.e. at first cycle) Capecitabine/5FU dose, and the last column facilitates comparability with the No-ToxNav cohort. There were 4 detected variants in the tested population. Two of them were classified as HFS (i.e. rs1213215 and rs2612091) and the remaining two were linked to critical risk (rs3918290) or high risk (rs67376798). Only one variant, which was related to HFS, was found in 2 tested patients of Asian background and 1 of African background. The distribution of the variants appeared to be similar across the different tumour sites. The missing observations for the dose of Capecitabine and 5FU were 20% in both ToxNav and No-Toxnav cohorts.

**Table 2:** ToxNav® variant results across patient characteristic and initial chemotherapy dose

	Standard (***n =*** 139)	rs12132152 HFS (***n =*** 31)	rs2612091 HFS (***n =*** 280)	rs3918290 Critical (***n =*** 7)	rs67376798 High(***n =*** 9)	No-ToxNav (***n =*** 1556)
	Count (column %, row %)	Count (column %, row %)	Count (column %, row %)	Count (column %, row %)	Count (column %, row %)	Count (column %)
Result classification*						
HFS	0 (0%)	31 (100, 10%)	280 (100, 90%)	0 (0%)	0 (0%)	NA
High/critical toxicity	0 (0%)	0 (0%)	0 (0%)	7 (100, 44%)	9 (100, 56%)	NA
Standard	139 (100, 100%)	0 (0%)	0 (0%)	0 (0%)	0 (0%)	NA
Race						
Caucasian	91 (65, 30%)	24 (77, 8%)	179 (64, 59%)	4 (57, 1%)	6 (67, 2%)	1079 (69%)
Asian	4 (3, 67%)	0 (0, 0%)	2 (1, 33%)	0 (0, 0%)	0 (0, 0%)	28 (2%)
African	1 (1, 50%)	0 (0, 0%)	1 (0, 50%)	0 (0, 0%)	0 (0, 0%)	15 (1%)
Other/unknown	43 (31, 28%)	7 (23, 5%)	98 (35, 64%)	3 (43, 2%)	3 (33, 2%)	3 (0%)
Tumour site						
Upper GI	19 (16, 27%)	4 (17, 6%)	45 (19, 63%)	1 (20, 1%)	2 (33, 3%)	299 (19%)
Lower GI	53 (45, 32%)	8 (33, 5%)	101 (42, 60%)	2 (40, 1%)	3 (50, 2%)	724 (47%)
Breast	35 (30, 30%)	8 (33, 7%)	71 (30, 61%)	2 (40, 2%)	1 (17, 1%)	382 (25%)
Other	10 (9, 29%)	4 (17, 11%)	21 (9, 60%)	0 (0, 0%)	0 (0, 0%)	132 (8%)
Initial Capecitabine dose*						
< 60%	0 (0, 0%)	0 (0, 0%)	4 (2, 50%)	2 (67, 25%)	2 (50, 25%)	36 (5%)
60–80%	7 (10, 21%)	1 (5, 3%)	25 (14, 74%)	1 (33, 3%)	0 (0, 0%)	120 (15%)
80–100%	60 (90, 27%)	19 (95, 8%)	144 (84, 64%)	0 (0, 0%)	2 (50, 1%)	630 (80%)
Initial 5FU dose*						
< 60%	5 (11, 45%)	0 (0, 0%)	2 (3, 18%)	1 (100, 9%)	3 (100, 27%)	27 (5%)
60–80%	5 (11, 31%)	2 (40, 13%)	9 (14, 56%)	0 (0, 0%)	0 (0, 0%)	69 (12%)
80–100%	34 (77, 38%)	3 (60, 3%)	52 (83, 58%)	0 (0, 0%)	0 (0, 0%)	461 (83%)

* *p-*value< 0.05 based on Chi-square test; Note: patients who received Capecitabine or 5FU prior to ToxNav test are excluded from the dose related variables displayed in this table; Initial dose refers to dose at the first cycle of treatment; rs3918290 and rs67376798 variant alleles were all heterozygote. NA: Not applicable

**Fig. 1:**
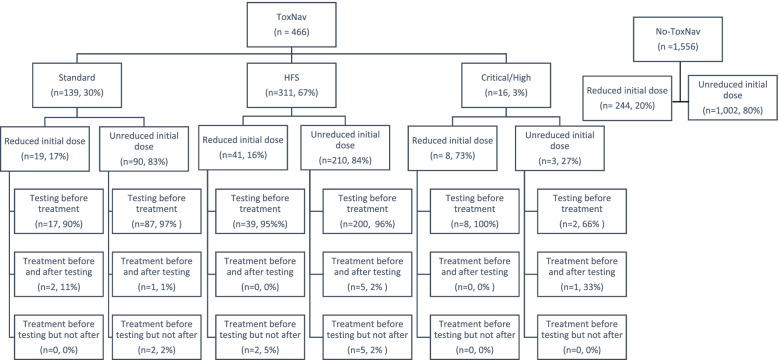
Flow chart of ToxNav® tested patients. Note: There were missing observations in the dose variable; Reduced initial dose was defined as less than 80% dose at first chemotherapy cycle; The proportion of reduced initial dose in the No-ToxNax cohort was added in the Figure for comparison purposes

There were 10 patients who had Capecitabine before ToxNav® testing, of whom 1 (1%) was later tested as Standard, 8 (80%) with HFS, and 1 (1%) with High/Critical toxicity (Fig. 1). All of them, except for the one without a variant, were given full dose Capecitabine at the beginning of their treatment. However, the dose was reduced to 50% for the patient with High/ Critical toxicity variant by the end of the treatment while, substantial dose reductions (i.e. to 75 and 50% dose) were observed in 4 (50%) patients of those with HFS. Similarly, there were 9 patients who had 5FU before ToxNav® of whom 5 (56%) were tested as HFS and 4 (44%) as Standard. With the exception of one patient who was given 75% dose of 5-FU, nearly all patients with HFS were given a full dose 5FU which in most cases (80%) was reduced to 75% by the end of the treatment.

There were a number of instances where clinicians went against the ToxNav® testing recommendations in terms of drug administration and dosing. For example, despite ToxNav® testing, there were 4 patients who did not receive Capecitabine and 5 patients who did not receive 5FU, but none of them were classified as High or Critical risk based on the test results. As shown in Table 2, one patient (33%) with the critical-risk variant (rs3918290) received only moderately reduced initial dose of Capecitabine (i.e. 60–80%) rather than no drug or 50% dose reduction, and 2 patients (50%) with the High-risk variant (rs67376798) received 100% initial dose of the same drug, rather than a reduced dose. In contrast, there were 30 patients with a HFS related variant who had reduced initial dose of Capecitabine despite the manufacturer’s recommendation to administer 100% dose for those variants. Similar proportion of noncompliance to the manufacturer’s recommendation were observed in the initial dosing of 5FU in patients with a HFS variant. Consistent with the recommendations, none of the patients with the High-risk variant was given higher than 60% initial dose of 5FU. Please see Appendix E, F and G, for more details on the dose, toxicity, and chemotherapy related mortality of patients who started chemotherapy before getting tested and patients with High and Critical variants.

One consideration for mandatory variant testing before drug administration is the potential for treatment delays. The time interval between prescription and administration (i.e. cycle one) of Capecitabine was 5.6 (SD: 13.8) days in the ToxNav cohort and 12.8 (SD: 35.8) days in the No-ToxNav cohort, resulting in a difference of 7.2 (95%CI: 4.3–10.1) days less waiting in the Toxnav cohort. Conversely, for those receiving 5FU, patients in the No-ToxNav cohort waited 3.1 (95%CI: 1.1–5.2) days less than in the ToxNav group (mean waiting days in that cohort: 4.9, SD: 11.6).

**Fig. 2:**
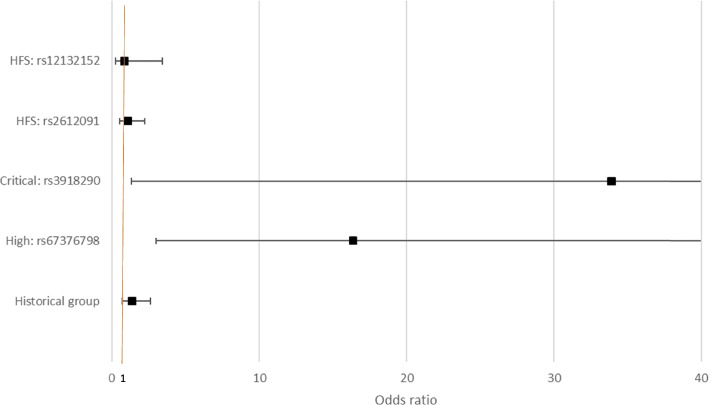
Likelihood of having a reduced initial dose of Capecitabine or 5FU compared to patients without a variant (after adjusting for confounding)

The likelihood of having a reduced (i.e. less than 80%) initial dose (i.e. at first cycle) of Capecitabine or 5FU was significantly higher in patients tested with the critical (OR: 33.93; 95%CI: 1.34–856.16) and high (OR: 16.40; 95%CI: 3.01–89.31) risk variants compared to patients without variants detected (Fig. 2). Importantly, the addition of the HFS variants in the ToxNav® variant panel resulted in no differences in the initial dose of either 5-FU or Capecitabine in patients with HFS variants compared with patients in the historical No-ToxNav group and patient without variants (standard risk).

### ToxNav® results and their impact on hospital costs

**Fig. 3:**
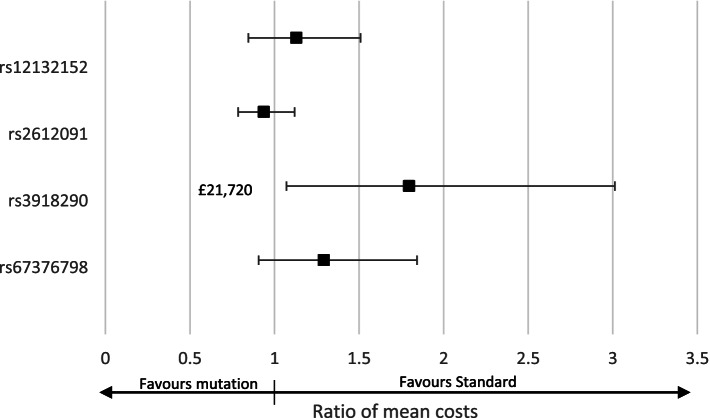
Differences in hospital costs of patients with *DPYD* variants compared to patients without a variant (after adjusting for confounding)

As Fig. 3 shows, patients with the Critical risk variant (rs3918290) had on average 80% (95%CI: 7–300) or £21,720 higher hospital costs than patients without a variant after adjusting for the confounders included in the PSM. This is reflected in the clinical outcomes for patients reported in Appendix F. Patients with the remaining variants did not have statistically significant different hospital costs than patients without a variant.

**Fig. 4:**
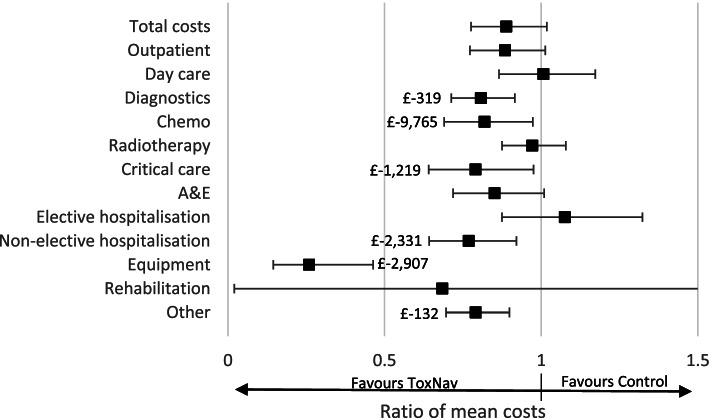
Impact of ToxNav® testing on average hospital costs per patient

ToxNav testing also appeared to reduce the cost of chemotherapy by 12% (95%CI: 3–31) or £9765, the cost of non-elective hospitalisation by 23% (95%CI: 8–36) or £2331, and the cost of critical care by 21% (95%CI: 2–36) or £1219 per patient. We also found reduced diagnostic costs (£319), equipment (£2907), and other costs (£132). As Shown in Fig. 4, the reduction in total hospital costs was borderline overall and not statistically significant.

### Impact of ToxNav® on drug adverse effects and patient reported depression scores

**Fig. 5:**
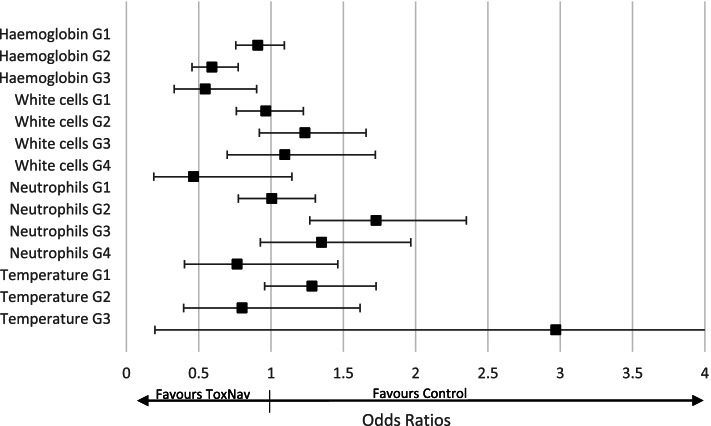
Impact of ToxNav on adverse events compared with No-ToxNav

The reporting of toxicity in this real-world cohort was limited to regular full blood count and temperature, with inconsistent reporting of symptomatic toxicity such as diarrhoea, mucositis and hand foot syndrome. Following evaluation of longitudinal full blood count testing, patients with ToxNav testing appeared to reduce the likelihood of experiencing moderate Grade 2 (OR: 0.59; 95%CI: 0.45–0.77) and severe anaemia Grade 3 (OR: 0.55; 95%CI: 0.33–0.90), while it increased the likelihood of mild neutropenia Grade 1 (OR: 1.73; 95%CI: 1.27–2.35) (Fig. 5). More detailed results are presented in Appendix H.

**Fig. 6:**
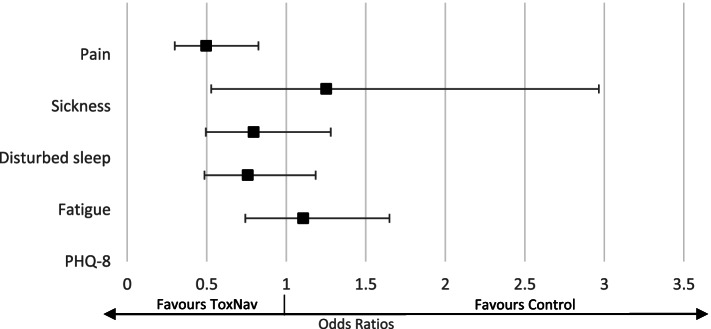
Impact of ToxNav on factors related to depression (as measured by the PHQ-8)

Figure 6 shows that patients tested with ToxNax® were less likely (OR: 0.50; 95%CI: 0.30–0.83) to report pain for more than 4 days a week than those patients not tested. No differences were found in terms of symptoms of sickness, disturbed sleep, and fatigue.

### Impact of ToxNav® on mortality

We did not specifically seek to assess the impact of on mortality ToxNax®, but assessment of the deaths associated with treatment in the ToxNav and Non-ToxNav groups (Table 1, Appendix G) suggests that non-cardiac deaths may be reduced in relative terms by ToxNax® panel testing. This clearly requires future longer term and expanded prospective cohorts in order to fully evaluate with stratification by variant.

## Discussion

This study provides evidence from a single centre real-world setting that upfront screening for the DPYD variants using ToxNav® leads to substantial hospital cost savings per cancer patient treated with Capecitabine and 5-FU. These savings were mainly driven by the reduction in the costs of non-elective hospital admissions and chemotherapy in patients who have high and critical risk DPYD variants. This is consistent with the use of test to provide personalised chemotherapy treatment that is effectively averting toxicity side effects in a minority of patients (1–2%) while also reducing the cost of chemotherapy. Despite a wider panel of variants in ToxNav®, we only detected 4 of the 19 variants covered by the test in our mainly Caucasian population. Importantly, despite no formal data collection for the severity of drug induced HFS, we did not observe impact of the commoner variant alleles associated with HFS in ToxNav® in terms of toxicity and costs.

The cost savings of ToxNav® are expected to largely outweigh the ToxNav® test price, which is expected to be in the region of £100–£300 per patient, and are in line with findings from previous studies on upfront screening for DPYD variants. A small single centre cost analysis in Ireland estimated the return on investment of implementing DYPD screening on a routine basis to be 600% (i.e. the cost savings through the prevention of unexpected hospital admissions for severe toxicity from fluoropyrimidine were 6 fold of the cost of prospectively testing at a €177 test price) [25]. A Dutch study of upfront screening for the four CPIC DPYD variants estimated a net cost saving of €51 per patient [9]. Cost savings were also reported when testing for 3 DPYD variants [7] or even just one [8]. In our case, the hospital overall costs were not significantly different between the ToxNav tested cohort and the Non-ToxNav historical controls, despite a significant cost improvement of over £20,000 associated with patients with high and critical risk variants. A larger pool of patients with high and critical variants may be needed (i.e. test a larger patient sample), including additional ethnicity associated variants, to observe a difference in overall hospital costs.

Although health outcomes were not included in our data, the results point to the direction of quality of life gains due to ToxNav®testing. The estimated reduction in the likelihood of experiencing haemoglobin grade 2 and grade 3 side effects could be translated into avoided disutility (i.e. reduction in health related quality of life) related to these side effects, which is − 0.17 -0.19 respectively on the EQ-5D scale (i.e. from 0-death to 1-perfect quality of life). These gains are unlikely to be outweighed by the estimated increased likelihood of neutrophils grade 2 side effect as its associated disutility is − 0.10 on the EQ. 5D scale. The estimated increased likelihood of neutrophils grade 2 side effect may be explained by differences in the timing of neutrophil testing as in the ToxNav group clinicians may were more vigilant about potential side effects. However, the mean number of neutrophil tests per patient was not statistically different between the two groups after adjusting for confounding. Furthermore, our results regarding reduced pain experience reinforce the argument that ToxNav may have a positive impact on quality of life. This expectation is in line with findings from a previous study that found gains in quality adjusted life years (QALYs) DPYD-guided toxicity management in cancer patients [12].

As there was an associated reduction in non-elective care costs, ToxNav® may also have prevented a proportion of the non-cardiac chemotherapy related deaths through better toxicity management. As our results show, non-cardiac deaths account for 73% of all chemotherapy related deaths. Applying this proportion to the 1200 deaths annually in the UK due to toxicity to Capecitabine and 5FU, a maximum number of 876 deaths could be avoided with upfront DPYD variant testing. This potential impact on mortality due to toxicity should be further investigated and considered by policy makers alongside the potential impact on patient’s quality of life and hospital costs.

Moreover, our finding that patients with the Critical 0–50% DPYD activity variant (i.e. rs3918290) had higher hospital costs compared to those without any tested variant, indicates that the test itself cannot reduce side effects and costs if it is not followed by appropriate Capecitabine/5-FU dose management. We observed a substantial number of cases were the manufacturer’s recommendations were not followed in real-world clinical decision-making. For example, there is no clear explanation about the reduced initial dose of Capecitabine/5-FU in some patients with a HFS variant other than clinical risk-aversion [26]. Defining and administering optimal dose levels of these drugs for a single or combination of DPYD variants is crucial in maximising the potential cost-effectiveness of ToxNav®. To fully evaluate the impact of the test on subsequent patient dose management and clinical outcomes, larger prospective studies are required in order to further develop variant specific stratification.

There is also an ongoing debate about what variants should be tested, as there is no evidence about what combination of tested variants would maximise the cost-effectiveness of upfront DPYD testing. In our Caucasian population, only two of the three CPIC variants were detected (without testing for HAPB3 in the panel), and a further two variants associated with HFS. Following the example of the Netherlands, NHS England is adopting the CPIC four variant testing through seven NHS Genome Laboratory Hubs (GLH) across England. However, the four CPIC variants are predominantly found in Caucasian populations, where they have been internally validated, and African variants are less represented in the GLH test. This of course raises ethical questions about health inequality but it also limits the potential cost savings of upfront DYPD testing as some DPYD variants are more prevalent to populations of African descent [15]. ToxNav®, and testing panels that are also broader and validated, could overcome such a selective testing and reduce unequal distribution of toxicity burden among cancer patients of different ethnic background. This should be seriously taken into consideration in the roll-out of upfront DPYD testing across Europe and further real-world evidence should be collected about the variants and the associated side effects and costs.

The strengths of this study include the relatively large sample of tested patients, the linked and detailed data on variants, treatment, and hospital costs. The main limitations are the imperfect recording of side effects, especially diarrhoea and mucositis, in the hospital setting and the subjectivity of the results to residual confounding although this is expected to be limited due to the effective matching of the two cohorts. Further limitations are the large number of missing observation in the doses of Capecitabine and 5FU but this may have a limited impact on the robustness of the results as the missingness was 20% in both ToxNav and No-ToxNav groups assuming missing completely at random (i.e. no systematic differences between missing and observed doses of chemotherapy). Perhaps a more significant omission is the lack of the **rs56038477/rs75017182** c1129–5923 C > G *DPYD* variant known as HAPB3. This occurs at relatively high frequency in Caucasian populations, meaning that it may have reduced the overall frequency of homozygote variant alleles, and so increase the propensity for patients testing positive with high and critical apparently heterozygous variants having a higher dose of 5-FU/ capecitabine.

## Conclusion

Upfront testing of DPYD variants with ToxNav® appears to reduce the toxicity burden of Capecitabine and 5-FU in cancer patients and can lead to substantial hospital cost savings depending on the variant. These savings are only captured if the dose management of the drugs in response to variants detected is standardised and regulated.

## Supplementary Information

**Additional file 1.**

## Data Availability

The data was obtained as part of a clinical audit in OUHFT that prohibits using or sharing the data beyond this study. For data enquiries please contact the corresponding author.
