# Supplementary material for: Can upfront DPYD extended variant testing reduce toxicity and associated hospital costs of fluoropyrimidine chemotherapy? A propensity score matched analysis of 2022 UK patients
Source: BMC Cancer. 2022 Apr 26;22:458. doi: 10.1186/s12885-022-09576-3 (PMC9044697; doi:10.1186/s12885-022-09576-3)
Supplement: Supplementary file 1 — Additional file 1. [file 12885_2022_9576_MOESM1_ESM.docx]

# Appendices

## ***Appendix A Regimen in the dataset***

|  | Toxnav | No-ToxNav |
| --- | --- | --- |
| 5FU(96hr CIV)withRT= | 1 (0.06%) | 1 (0.21%) |
| ACTICCA-1 = | 0 0 | 1 0.21 |
| Aristotle cape irin= | 1 0.06 | 0 0 |
| CF (75) Infusor= | 3 0.19 | 0 0 |
| CF (80) Infusor= | 16 1.03 | 5 1.07 |
| CF Infusor (SCC)= | 1 0.06 | 0 0 |
| CF Infusor= | 7 0.45 | 3 0.64 |
| CHARIOT Cispl Capec= | 5 0.32 | 0 0 |
| Cape 5d +RTpancr= | 8 0.51 | 4 0.86 |
| Cape Mitomycin RT= | 51 3.28 | 19 4.08 |
| Capecitabine 5d RT= | 173 11.12 | 74 15.88 |
| Capecitabine 7/7= | 10 0.64 | 0 |
| Capecitabine Adj CR= | 31 1.99 | 11 2.36 |
| Capecitabine BILCAP= | 0 | 1 0.21 |
| Capecitabine Met CR= | 14 0.9 | 3 0.64 |
| Capecitabine-1000= | 1 0.06 | 0 |
| Carbo 5FU CrCl infu= | 1 0.06 | 0 |
| Carbo Cape CrCl= | 1 0.06 | 0 |
| Cetuximab IrMdG= | 4 0.26 | 1 0.21 |
| Cisp Doce Fluor TPF= | 2 0.13 | 0 |
| Cisplatin Cape Anal= | 1 0.06 | 0 |
| Cisplatin Cape= | 29 1.86 | 5 1.07 |
| Cisplatin Capecitab= | 15 0.96 | 3 0.64 |
| Cisplatin(100)RTday= | 1 0.06 | 0 |
| Cisplatin(40)RT Day= | 3 0.19 | 1 0.21 |
| Docet Oxalip Flurou= | 8 0.51 | 8 1.72 |
| EC= | 2 0.13 | 3 0.64 |
| ECCapecitabineDaypt= | 22 1.41 | 0 |
| ECF Daypt= | 4 0.26 | 1 0.21 |
| EO-Capecitabine= | 48 3.08 | 14 3 |
| EO-Fluorouracil= | 5 0.32 | 0 |
| ESPAC 4 Gem Cap= | 1 0.06 | 0 |
| Epirubicin-20= | 1 0.06 | 0 |
| FEC 75/500= | 21 1.35 | 0 |
| FEC Doc Pert Tra ad= | 8 0.51 | 0 |
| FEC Doce Pertu Tras= | 42 2.7 | 1 0.21 |
| FEC docetaxel= | 303 19.47 | 7 1.5 |
| FEC-100= | 10 0.64 | 1 0.21 |
| FOLFIRINOX adjuvant= | 2 0.13 | 7 1.5 |
| FOLFIRINOX= | 49 3.15 | 18 3.86 |
| FOLFOXIRI= | 1 0.06 | 2 0.43 |
| GO2 OxCap 100%= | 1 0.06 | 0 |
| GO2 OxCap 60%= | 1 0.06 | 0 |
| Gem-Cis-ABC-Daypt= | 1 0.06 | 4 0.86 |
| Gem/CarboCrClBreast= | 7 0.45 | 4 0.86 |
| GemCape-Pancreatic= | 10 0.64 | 1 0.21 |
| GemCis1000/70 outpt= | 1 0.06 | 0 |
| Gemcarb(CrCl)1000= | 1 0.06 | 0 |
| Gemcitabine Abraxan= | 2 0.13 | 0 |
| Gemcitabine-Panc= | 4 0.26 | 3 0.64 |
| InterAACT cis-5fu= | 1 0.06 | 0 |
| Irin-ModDeGramont= | 115 7.39 | 4 0.86 |
| Irino ModDeGra(NET)= | 1 0.06 | 0 |
| Irinotecan + cape= | 3 0.19 | 1 0.21 |
| Irinotecan= | 1 0.06 | 0 |
| LUD2015-005 A1,A2,C= | 10 0.64 | 2 0.43 |
| LUD2015-005 A1,C,D= | 2 0.13 | 0 |
| LUD2015-005 C-FLOT= | 1 0.06 | 2 0.43 |
| LUD2015-005 CohortC= | 2 0.13 | 1 0.21 |
| LUD2015-005 Run B= | 1 0.06 | 0 |
| LUD2015-005 post= | 4 0.26 | 1 0.21 |
| LUD2015-005 run A2= | 2 0.13 | 0 |
| MF+RT<=70 infusor= | 2 0.13 | 1 0.21 |
| Oxal Cape= | 194 12.47 | 85 18.24 |
| Oxal-ModDeGramont= | 103 6.62 | 47 10.09 |
| PARTNER FEC100= | 22 1.41 | 3 0.64 |
| PLATFORM Arm A1= | 1 0.06 | 0 0 |
| PLATO Mito Cap 28d= | 10 0.64 | 8 1.72 |
| PLATO Mito Cape 23d= | 4 0.26 | 3 0.64 |
| PRIMUS 001 Gem PacA= | 0 0 | 1 0.21 |
| Panitu Irin ModDeGr= | 0 0 | 2 0.43 |
| Panitu Oxal ModDeGr= | 0 0 | 2 0.43 |
| Raltitrexed+oxalip= | 0 0 | 1 0.21 |
| SCALOP-2 GEMABX= | 3 0.19 | 1 0.21 |
| SCALOP-2 Nelf Cape= | 1 0.06 | 0 0 |
| SCOPE 2 C2-4 Arm2+4= | 2 0.13 | 1 0.21 |
| SCOPE 2 cycle 1 all= | 1 0.06 | 1 0.21 |
| STAR-TREC= | 1 0.06 | 0 |
| Streptozocin+cape= | 11 0.71 | 0 0 |
| TULIP Lapatin Capec= | 1 0.06 | 0 0 |
| TrastuzumabCispCape= | 20 1.29 | 9 1.93 |
| capecitabine 1000= | 79 (5.08 | 19 (4.08) |
| Missing | 24 (1.54) | 65 (13.95) |
| Total | 1,556 (100%) | 466 (100%) |

***Appendix B ToxNav Test***

Out of the four CPIC variants, two *DPYD* minor variant alleles can cause severe side effects (**rs3918290** c1905+1G>A known as *DPYD* 2A* MVAF 1.6% enzyme activity 0%, and **rs55886062** c1679 T>G known as DPYD*13 pI560S MVAF 0.01% enzyme activity 20%) and usually mean the patient with the former allele should not receive either capecitabine or 5-FU if they have varaints in both alleles (see Table below). It is frequently one of these low enzyme activity variants that accounts for post-treatment deaths in 0.1-1% of all patients treated. The remaining two variants have intermediate enzyme activity, and doses are normally reduced rather than treatment being stopped (**rs67376798** c2846A>T DPYD pD949V MVAF 0.7% enzyme activity 50%, **rs56038477/rs75017182** c1129-5923 C>G known as HAPB3 MVAF 4% enzyme activity 75%).

There are differences between the CPIC guidelines for dose adjustment and those associated with ToxNav mainly in relation to the omission of the HAPB3 higher frequency (4%) variant allele with moderate impact on DPYD enzyme activity (Amstutz et al 2017). The CPIC guidelines assign a metaboliser activity score based on the variant and whether either one (heterozygous) or two alleles (homozygous) are affected. The AS scores range from 2 (normal or wild-type), 1-1.5 (intermediate) to 0-5 (poor). The clinical dosing of 5-FU and capecitabine is matched to the AS score, with no change for AS=2, dose reduction of 50% for AS=1 and 25% for AS=1.5 with preferable avoidance or extremely low doses of the use of 5-FU/ capecitabine with AS=0.5, and complete avoidance recommended with AS=0. To be specific, for the critical DPYD variants (**rs3918290** c1905+1G>A known as DPYD 2A* MVAF 1.6% enzyme activity 0% and **rs55886062** c1679 T>G known as DPYD*13 pI560S MVAF 0.01% enzyme activity 20%), the advice is the same between CPIC and ToxNav if the AS score is 0-0.5 (with homozygous variants), and that is to avoid 5-FU/capecitabine and use an alternative. For the intermediate AS activity, homozygous intermediate activity variants (e.g. **rs67376798** c2846A>T DPYD pD949V MVAF 0.7% enzyme activity 50%) as well as heterozygous critical variants (e.g. **rs3918290**) warrant dose reductions of at least 50% are advised in both CPIC 2017 and ToxNav for heterozygous critical DPYD variants alone, and when there are homozygous intermediate activity alleles. Overall, the ToxNav interpretation will underestimate the dose reductions needed as the high frequency HAPB3 allele is not assessed, leading to homozygous variants being underestimated and dosing being higher in some cases.

ToxNav is based on a prospective and unbiased analysis of toxicity in the Quasar 2 (Quick and Simple and reliable 2, randomised adjuvant chemotherapy study in colon and rectal cancer) clinical trial of 888 patients receiving capecitabine chemotherapy, resulted several known and new variants in DPYD and in the ENOSF1 gene (Enolase Superfamily Member 1) being identified.^1 2^ The latter was associated with the development of HFS toxicity.^3^ In addition, microRNAs such as miR27a and miR 27b have been shown to repress DPYD, and are also associated with capecitabine and 5-FU toxicity. A small study of 60 patients with colorectal cancer (PRECISE) prospectively evaluated sensitivity and specificity of an extended panel of variant alleles using patient reported toxicity testing.^4^

Supplementary Table 1 Toxnav test risk classification

| **Patient Classification** | **Alleles** | **Predicted DYPD enzyme activity** | **Recommended Dosage of 5-FU/ capecitabine** | **Key variants** |
| --- | --- | --- | --- | --- |
| Critical risk | Homozygous | 0-50% | Avoid dosing | rs3918290/ rs3918290  rs3918290/intermediate  rs55886062/ rs55886062  rs55886062/intermediate |
| Critical risk | Heterozygous | 50% | 25-50% cautious dosing | rs3918290/ +  rs55886062/ + |
| High risk | Homozygous | 50% | 50% dosing | rs67376798/ rs67376798 |
| High risk | Heterozygous | 75% | 50% dosing and titration | rs67376798/+ |
| HFS risk | Heterozygote | >80% | 100%  Hand foot risk | rs12132152/+  rs2612091/+ |
| Normal risk | Wild-type | 100% |  | +/+ |

***Appendix C Statistical Analysis***

We adopted a step-wise strategy to select the propensity score matching (PSM) technique that most reduced observed confounding between the two cohorts.^5-8^ First, we included all possible confounding variables in the dataset, and considered that the inclusion of covariates not associated with the treatment assignment would have little influence in the propensity score model.^5^ Second, we matched the two cohorts using a range of the most commonly used PSM techniques; these included Mahalanobis, 1-to-1, K-to-1, kernel, local linear regression, spline, and inverse probability weighting techniques. Second, the performance of each PSM technique on covariate balancing was assessed based on the mean and median percentage standardised bias as well as Rubin’s B (the absolute standardized difference of the means of the linear index of the propensity score in the treated and (matched) non-treated group) and Rubin’s R (the ratio of treated to (matched) non-treated variances of the propensity score index). Following Rubin’s (2001) recommendation, we considered B less than 25 and R between 0.5 and 2 to indicate sufficient balance.^9^ Third, we chose the PSM technique that had the lowest values on these performance indicators. We matched the two cohorts by socio-demographic characteristics (i.e. age, gender, ethnicity), diagnosis code (i.e. primary ICD-10 code), treatment (i.e. regimen and number of chemotherapy cycles), duration of follow-up (i.e. the interval between June 2017 and either September 2020 or the date of death), and estimated survival based on all other observed confounders. The later was included in the PSM as a proxy of cancer severity.

We calculated means, standard deviations (SDs) and frequencies to describe differences in patient characteristics and tested differences using a two sample t-test and Mann-Whitney test for continues variables and χ2 test for categorical variables. Furthermore, we performed regression analysis using generalised linear regression models (GLMs) with gamma distribution and log link to compare the hospital costs associated with the different mutations with the hospital costs of those without found mutations after adjusting for the confounders mentioned above. ^10^

Moreover, we performed a doubly robust estimation to further reduce confounding by using a regression analysis after performing the most suitable PSM technique and including the confounding variables listed above as covariates.^10^ In the regressions, we used GLMs with gamma distribution and log link to investigate the association of ToxNav with hospital costs during the follow-up period. We also used GLMs with binomial distribution and logit link to estimate the odds ratios (OR) of experiencing adverse events from chemotherapy as well as psychological outcomes. For each adverse event, we calculated the percentage of each grade (i.e. level of severity) out of all observations per patient. This percentage was included as a dependent variable in the regressions. For psychological effects, we calculated the percentage of all observations per patient with a symptom frequency higher than 3 days a week and the PHQ-8 score higher than 10 (i.e. moderate to severe depression). Robust standard errors were specified in all regression models. There were few missing observations in the dataset and thus, complete case analysis was performed. We also performed a regression analysis with an interaction term of the ToxNav dummy and the dose reduction variable to investigate the association between hospital costs and dose reduction in the two cohorts.

## ***Appendix D Covariate balance after propensity score matching***

| Variable | Matched | Treated | Control | %bias | bias | t | p>t | V(C) |
| --- | --- | --- | --- | --- | --- | --- | --- | --- |
| Age at start of FU | U | 61.27 | 60.062 | 9.7 |  | 1.8 | 0.072 | 0.88 |
|  | M | 61.069 | 59.576 | 11.9 | -23.6 | 1.59 | 0.113 | 0.86 |
| Gender | U | 1.5322 | 1.4216 | 22.3 |  | 4.23 | 0.000 | 1.02 |
|  | M | 1.5219 | 1.4969 | 5 | 77.5 | 0.67 | 0.501 | 1 |
| Ethnicity | U | 2.3433 | 2.1536 | 10.4 |  | 1.99 | 0.047 | 1.1 |
|  | M | 2.3443 | 2.4483 | -5.7 | 45.2 | -0.74 | 0.458 | 0.96 |
| ICD10 | U | 45.721 | 44.092 | 7.2 |  | 1.28 | 0.200 | 1.04 |
|  | M | 45.178 | 44.423 | 3.3 | 53.7 | 0.46 | 0.648 | 1.03 |
| Regimen | U | 49.993 | 47.513 | 9.1 |  | 1.7 | 0.089 | 1.35* |
|  | M | 49.751 | 51.499 | -6.4 | 29.5 | -0.82 | 0.413 | 1.05 |
| cycle_max_p | U | 3.8389 | 5.1122 | -21.7 |  | -3.18 | 0.001 | 0.14* |
|  | M | 3.7678 | 3.6528 | 2 | 91 | 0.49 | 0.621 | 0.73* |
| FU_months | U | 13.795 | 24.644 | -106.6 |  | -16.02 | 0.000 | 0.22* |
|  | M | 13.844 | 13.116 | 7.2 | 93.3 | 1.3 | 0.193 | 0.49* |
| Predicted mortality | U | 0.36625 | 0.26323 | 31.3 |  | 5.21 | 0.000 | 0.73* |
|  | M | 0.36271 | 0.40939 | -14.2 | 54.7 | -2.05 | 0.041 | 0.85 |

* if variance ratio outside [0.83; 1.20] for U and [0.81; 1.23] for M

| Sample | Ps R2 | LR chi2 | p>chi2 | MeanBias | MedBias | B | R | %Var |
| --- | --- | --- | --- | --- | --- | --- | --- | --- |
| Unmatched | 0.195 | 367.24 | 0 | 27.3 | 16 | 121.0* | 0.38* | 50 |
| Matched | 0.011 | 10.74 | 0.217 | 7 | 6 | 24.3 | 0.94 | 25 |

* if B>25%, R outside [0.5; 2]

**Supplementary figure 1 Standardised percentage bias before and after propensity score matching**

***Appendix E Analysis of ToxNAV Cohorts with DPYD mutation (Capecitabine before ToxNAV, 5FU before ToxNAV, both before ToxNAV)***

|  | **Patient** | **ToxNAV Variant (pre-chemo)** | **Drug 1** | **Drug 1 Toxicity** | **ToxNAV variant (post-drug 1)** | **Drug 2** | **Drug 2 Toxicity** | **ToxNAV variant (post-drug 2)** | **Drug 3** | **Drug 3 Toxicity** |
| --- | --- | --- | --- | --- | --- | --- | --- | --- | --- | --- |
| **Capec before ToxNAV** | 757 |  | Capecitabine 1g/m^2^ with Oxaliplatin 130mg/m^2^  *Completed 8 cycles* | G3 Cardiac (chest pain with hospital admission) after C1  *50% dose reduction* | HFS  rs12132152 | 5FU Irinotecan / Modified deGramont  *Nil administered* |  |  |  |  |
|  | 1212 |  | Capecitabine 625mg/m^2^, Oxaliplatin 130mg/m^2^, Durvalumab 750mg  *Completed 2 cycles* | None |  | Capecitabine 625mg/m^2^, Cisplatin 60mg/m^2^, RT  *Completed 2 cycles* | None | HFS  rs2612091 | Capecitabine 750mg/m^2^, Trastuzumab 8mg/kg, Cisplatin 60mg/m^2^  *Completed 2 cycles* | None |
|  | 1069 |  | Capecitabine 900mg/m^2^ with RT.  *Completed 1 cycle* | None | HFS  rs2612091 | Capecitabine 1g/m^2^ (C1) and 750mg/m^2^ (C2,3) with Oxaliplatin 130mg/m^2^ (C1) and 97.5mg/m^2^ (C2,3)  *Completed 3 cycles* | G1 diarrhoea after C1  *75% dose reduction* |  |  |  |
|  | 1800 |  | Capecitabine 823mg/m^2^  *Completed 1 cycle* | G3 Cardiac (chest pain with hospital admission) after C1  *Dose stopped* | STD |  |  |  |  |  |
|  | 477 |  | Capecitabine 1g/m^2^, Oxaliplatin 130mg/m^2^  *Completed 4 cycles* | G1 diarrhoea after C2  *No dose change* | HFS  rs2612091 | Capecitabine 900mg/m^2^, RT  *Completed 1 cycle* | None |  |  |  |
|  | 778 |  | Capecitabine 900mg/m^2^ with RT  *Completed 1 cycle* | G3 Cardiac (chest pain with hospital admission) after C1  *Dose stopped* | HFS  rs2612091  *50% dose reduction* | Capecitabine 500mg/m^2^, Oxaliplatin 104mg/m^2^  *Completed 1 cycle* | G3 Cardiac (chest pain with hospital admission)  *Dose stopped* |  |  |  |
|  | 1628 |  | Capecitabine 1mg/m^2^ with RT  *Completed 5 cycles* | G1 diarrhoea after C2 | HFS  rs2612091 | Oxaliplatin Modified DeGramont: 5FU 400mg/m^2^ IV once and 2,400mg/m^2^ IV with Oxiplatin 85mg/m^2^  *Completed 5 cycles* | None |  |  |  |
|  | 1638 |  | Capecitabine 625mg/m^2^, Cisplatin 60mg/m^2^ with RT  *Completed 2 cycles* | G3 diarrhoea and G1 HFS after C2  *9-day hospital admission.*  *Capecitabine stopped.* | HIGH  **rs3918290**  *50% dose reduction* | Capecitabine 312.5mg/m^2^, Oxaliplatin 130mg/m^2^, Epirubicin hydrochloride 50mg/m^2^  *Completed 1 cycle.* | None  (admitted to hospital for COPD exacerbation) |  |  |  |
|  | 468 |  | Capecitabine 900mg/m^2^ with RT  *Completed 1 cycle* | None |  | Capecitabine 1g/m^2^, Oxaliplatin 130mg/m^2^  *Completed 4 cycles* | G1 HFS after cycle 1 | HFS  rs2612091  *90% dose reduction* | Capecitabine 900mg/m^2^ with RT  *Completed 1 cycle* | None |
| **5FU before ToxNAV** | 1050 |  | Folfirinox: 5FU 1,800mg/m^2^ continuous and 300mg/m^2^ bolus, Oxaliplatin 63.75mg/m^2^, Irinotecan 135mg/m^2^  *Completed 4 cycles* | G1 diarrhoea after C1  *50% dose reduction* | HFS  rs2612091 | Capecitabine 622.5mg/m^2^, Gemcitabine 1g/m^2^  *Completed 1 cycle* | None |  |  |  |
|  | 1906 |  | Oxaliplatin Modified DeGramont: 5FU 400mg/m^2^ bolus and 2,400mg/m^2^ 46h infusion  *Completed 2 cycles* | None | STD | Capecitabine 625mg/m^2^, Oxaliplatin 104mg/m^2^, Epirubicin 50mg/m^2^  *Completed 5 cycles* | G1 diarrhoea after C2  G2 HFS after C4  *75% dose reduction* |  |  |  |
|  | 375 |  | Oxaliplatin Modified DeGramont: 5FU 400mg/m^2^ bolus and 2,400mg/m^2^ 46h infusion, Oxaliplatin 85mg/m^2^  *Completed 12 cycles* | None |  | Cetuximab Irinotecan Modified DeGramont: 5FU 400mg/m^2^ bolus and 2,400mg/m^2^ 46h infusion, Irinotecan 180mg/m^2^, Cetuximab 500mg/m^2^  *Completed 7 cycles* | None | HFS  rs2612091 | Capecitabine 900mg/m^2^ with RT  *Completed 1 cycle* | G1 HFS mid-cycle  *No dose change* |
|  | 145 |  | Oxaliplatin Modified DeGramont: 5FU 400mg/m^2^ bolus and 2,400mg/m^2^ 46h infusion, Oxaliplatin 85mg/m^2^  *Completed 12 cycles* | G1 HFS and diarrhoea post-C10.  (*80% dose reduction due to low neutrophils)* | HFS  rs2612091 | CEDAR Capecitabine RT (trial): Capecitabine 900mg/m^2^  *Completed 1 cycle*  *Dose reduction d22 (675mg/m^2^)* | None  *(75% capec dose reduction due to neutropenia)* |  |  |  |
|  | 210 |  | FEC (100) Docetaxel (100): 5FU 500mg/m^2^, Epirubicin 100mg/m^2^, Cyclophosphamide 500mg/m^2^  *Completed 6 cycles* | None | HFS  rs2612091 |  |  |  |  |  |
|  | 536 | STD | Folfirinox: 5FU 200mg/m^2^ bolus and 2,250mg/m^2^ 46h infusion, Oxaliplatin 85mg/m^2^  *50% 5FU dose reduction due to raised ALT.*  *Completed 5 cycles* | G1 diarrhoea  *Increased to 75% dose for C2 onwards (after discovering STD risk).* |  |  |  |  |  |  |
|  | 539 |  | 5FU 2g/m^2^ 96h infusion, Cisplatin 40mg/m^2^  *50% 5FU dose (to pre-emptively minimise toxicity)*  *Completed 2 cycles* | None | STD  *Screened post-C1* |  |  |  |  |  |
|  | 1652 |  | FEC (100) Docetaxel (100): 5FU 500mg/m^2^, Epirubicin 100mg/m^2^, Cyclophosphamide 500mg/m^2^  *Completed 6 cycles* | None | STD |  |  |  |  |  |
| **Both Capec and 5FU before ToxNAV** | 1536 |  | Capecitabine 1g/m^2^, Oxaliplatin 130mg/m^2^  *Completed 2 cycles* | None (only nausea) |  | Oxaliplatin Modified DeGramont: 5FU 2,400mg/m^2^ continuous over 46h and 400mg/m^2^ bolus, Oxaliplatin 85mg/m^2^  *Completed 5 cycles* | G1 diarrhoea after 5/3/20  *75% 5FU dose reduction (mainly due to nausea)* | HFS  rs2612091  *Identified after C3 Oxal Mod DeGramont* | 5FU 4g/m^2^ 96h infusor with RT  *Completed 1 cycle* | None |

**Appendix F Analysis of ‘High Risk’ ToxNAV Variant Patients**

| **Patient** | **ToxNAV Variant (pre-chemo)** | **Drug 1** | **Drug 1 Toxicity** | **ToxNAV variant (post-drug 1)** | **Drug 2** | **Drug 2 Toxicity** | **ToxNAV variant (post-drug 2)** |
| --- | --- | --- | --- | --- | --- | --- | --- |
| 491 | HIGH  rs67376798 | Mitomycin Capecitabine + RT: Mitomycin 12mg/m^2^, Capecitabine 825mg/m^2^  *Proceeded with full dose*  *Completed 1 cycle* | G1 HFS after d12  *No hospital admissions.*  *No dose change.* |  |  |  |  |
| 1559 | HIGH  rs67376798 | PLATO Mitomycin Capecitabine 28 days: Mitomycin 12mg/m^2^, Capecitabine 825mg/m^2^  *Proceeded with full dose treatment (bloods every 2d for week 1 and 2x per week for week 2)*  *Completed 25d.* | G2 diarrhoea and G1 HFS.  *1 day hospital admission.*  *Capecitabine stopped after 25d due to toxicity.* |  |  |  |  |
| 1638 |  | Capecitabine 625mg/m^2^, Cisplatin 60mg/m^2^ with RT  *Completed 2 cycles* | G3 diarrhoea and G1 HFS after C2  *9-day hospital admission.*  *Capecitabine stopped.* | HIGH  **rs3918290**  *50% dose reduction* | Capecitabine 312.5mg/m^2^, Oxaliplatin 130mg/m^2^, Epirubicin hydrochloride 50mg/m^2^  *Completed 1 cycle.* | None  (admitted to hospital for COPD exacerbation) |  |
| 1624 | HIGH  **rs3918290**  *50% dose reduction* | Capecitabine with RT (5 day): Capecitabine 450mg/m^2^  *Completed 1 cycle.* | G1 diarrhoea throughout treatment.  *No other issues/hospital admissions relating to treatment.* |  |  |  |  |
| 790 | HIGH  rs67376798  *50% dose reduction* | Capecitabine 500mg/m^2^, Oxaliplatin 130mg/m^2^  *Completed 3 cycles* | None |  | Panitumumab Irinotecan Modified DeGramont: Panitumumab 6mg/kg, Irinotecan 180mg/m^2^, 5FU 200mg/m^2^ bolus and 1,200mg/m^2^ over 46h  *50% 5FU dose reduction from C1.*  *Completed 2 cycles (stopped due to disease progression)* | None |  |
| 1106 | HIGH  **rs3918290**  *50% Capecitabine dose reduction* | Capecitabine 500mg/m^2^, Oxaliplatin 104mg/m^2^  *Completed 6 cycles* | G1 diarrhoea post-C2  *No other issues.* |  |  |  |  |
| 1289 | HIGH  **rs3918290**  *60% Capecitabine dose reduction* | Capecitabine 595.2mg/m^2^, Oxaliplatin 130mg/m^2^  *Completed 3 cycles* | G1 diarrhoea and HFS post-C1  *Admitted to EAU with UTI.*  *50% dose reduction after C2*  *G2 diarrhoea post-C3*  *Chemo stopped due to toxicities.* |  |  |  |  |
| 1555 | HIGH  rs67376798  *50% Capecitabine dose reduction* | Capecitabine 500mg/m^2^, Oxaliplatin 130mg/m^2^  *Completed 4 cycles* | None |  |  |  |  |
| 1597 | HIGH  **rs3918290**  *50% 5FU dose reduction* | Oxaliplatin Modified DeGramont: Oxaliplatin 42.5mg/m^2^, 5FU 200mg/m^2^ bolus and 1,200mg/m^2^ 46h infusion  *Completed 1 cycle.*  *Admitted to hospital with fever 5d post-C1 (cellulitis on top of lymphoma, not neutropenic).*  *Chemo stopped.* | None |  |  |  |  |
| 1853 | HIGH  rs67376798  *50% 5FU dose reduction* | Oxaliplatin Modified DeGramont: Oxaliplatin 63.75mg/m^2^, 5FU 200mg/m^2^ bolus and 1,200mg/m^2^ 46h infusion  *Completed 7 cycles* | *Dose reduction to 40% pre-C7 due to mouth ulcers. No other chemo-associated toxicities.*  *Doses stopped after C7 due to disease progression.* |  |  |  |  |
| 1939 | HIGH  rs67376798  *Excluded 5FU from C1.*  *25% dose in C2.* | ECF Daypt: Epirubicin 37.5mg/m^2^, Cisplatin 60mg/m^2^, 5FU 1,050mg/m^2^  *Completed 2 cycles* | *5FU stopped due to neutropenia.* |  |  |  |  |
| 14466 | HIGH  rs67376798  *50% Capecitabine dose reduction* | EC Capecitabine Daypt: Epirubicin 50mg/m^2^, Cisplatin 60mg/m^2^, Capecitabine 312.5mg/m^2^  *Completed 6 cycles.* | Chronic SOB (6-9 months, pre-chemo) induced by minimal exertion. Investigated in hospital post-C6. Unknown cause.  *Chemo stopped due to the above and no active cancer progression.* |  |  |  |  |
| 14598 | HIGH  rs67376798  *Decision to use another drug regimen not involving 5FU or Capecitabine due to age of patient (75y) and high risk.* | Paclitaxel 175mg/m^2^, Carboplatin 450mg (at 5 AUC CrCl), Trastuzumab 8mg/kg  *Dose reduction after C1 due to poor renal function.*  *Completed 2 cycles.*  *Treatment stopped due to disease progression.* | No significant toxicity relating to chemotherapy.  *Admitted to hospital with progressive disease, fever, vomiting* |  |  |  |  |
| 15154 | HIGH  **rs3918290**  *Decision not to start chemotherapy (patient not being able to access help if needed).* | No chemotherapy administered. |  |  |  |  |  |
| 15567 |  | Trastuzumab emtansine (Kadcycla): 3.6mg/kg  (20/2/19)  *Completed 22 cycles*  SYD985.002 (TULIP) SYD985 arm: SYD985 1.2mg/kg (21/7/20)  *Completed 7 cycles*  Denusumab 120mg  *Completed 14 cycles* | Persistent neutropenia | HIGH  **rs3918290**  *Never started on 5FU or Capecitabine* | Epirubicin 75mg/m^2^, Cyclophosphamide 500mg/m^2^ (28/12/20)  *Completed 3 cycles* | Significant neutropenic sepsis post-C1, G2 diarrhoea.  *20% dose reduction for C2, but never given.*  *Admitted to hospital with fever. Stayed for 16 days then passed away.* |  |
| 15609 | HIGH  rs67376798  *Never started on 5FU or Capecitabine.* | Gemcitabine 800mg/m^2^  *80% dose reduction due to raised bilirubin.*  *Completed 1 cycle.* | Low platelets post-C1.  *C1 D8 delay and dose reduction to 75%*  *Admitted to hospital post-stroke. No further chemotherapy as too unwell.* |  |  |  |  |

**Appendix G Analysis of Deaths within 30 days**

No. Patient Deaths within 30d

|  | **Without ToxNav prior to Rx** | **ToxNav screen prior to Rx (risk variant)** |
| --- | --- | --- |
| **Total number of deaths** | 36 | 7 |
| **Deaths due to Capecitabine or 5FU** | 10 | 1 (HFS) |
| **Deaths due to disease** | 23 | 6 (4 HFS, 2 STD) |
| **Deaths of unknown cause** | 3 | 0 |
| **Proportion of Capecitabine/5FU-induced deaths with cardiac association** | 0.2 | 1 |

Analysis of Deaths within 30 days related to Capecitabine or 5FU

| **Patient ID**  **(anonymised)** | **ToxNav variant** | **Agent** | **Time from last dose to admission** | **Toxicities** | **Length of hospital stay** | **Recorded cause of death** |
| --- | --- | --- | --- | --- | --- | --- |
| 75 | *not screened* | Capecitabine | 4 days | G3 diarrhoea, ?C. diff | 14 days | 1a. Metastatic breast cancer  2. Colitis (did not improve with IV fluids/Octreotide or Abx) |
| 1633* | *not screened* | Fluorouracil | 3 days | G3 neutropenic sepsis during C3 | 5 days | Chemotherapy-induced neutropenic sepsis and AKI. |
| 923 | *not screened* | Capecitabine | 12 days | G3 diarrhoea | 8 days | 1a. Intestinal perforation  1b. Chemotherapy for oesophageal carcinoma |
| 945 | *not screened* | Capecitabine | 6 days | G2 diarrhoea after C2, G3 cardiac (chest pain) prior to death. | *Died at home after chest pain in the night (28/11/2018).* | 1a. Metastatic adenocarcinoma of the oesophagus, Her2 positive |
| 121* | *not screened* | Capecitabine | 14 days | G2 diarrhoea, neutropenic sepsis. | 14 days | 1a. Capecitabine toxicity  1b. Rectosigmoid cancer |
| 1693* | *not screened* | Capecitabine | 4 days | Cardiac arrest | <24 hours | 1a. Cardiac toxicity associated with Capecitabine treatment  1b. Colonic cancer. |
| 1560* | *Retrospectively screened: partial DPD deficiency and early severe toxicity to fluoropyrimidine therapy (DYPD c.1679T>G and c2846A>T, p.D949V)* | Capecitabine | 21 days | G2 diarrhoea, G3 pancytopenia, panmucositis after C1. | <24 hours | 1a. Left lower lobe pneumonia  1b. Oral and GI mucositis induced by chemotherapy toxicity  2. Pulmonary embolism |
| 554* | *not screened* | Fluorouracil | 9 days | G2 diarrhoea, G3 neutropenic sepsis | 4 days | 1a. Viral pneumonia  1b. Chemotherapy  2. Pancreatic cancer |
| 731* | *not screened* | Capecitabine | 21 days | G3 dyspnoea, oedema, AKI, lung changes due to drug reaction.  *Prior to C4* | 3 days | 1a. Metastatic colorectal cancer |
| 992* | *not screened* | Fluorouracil | 2 days | G1 diarrhoea, G3 neutropenic sepsis due to CAP | <24 hours | 1a. Pneumonia  1b. Neutropenic sepsis  1c. Colorectal cancer |
| 1063 | HFS  rs2612091 | Fluorouracil | 6 days | Cardiac arrest. Died at home after ringing triage | <24 hours after call | ?Chemotherapy-induced Cardiac arrest |

* indicates patient deaths reviewed in M+M discussions

***Appendix H Impact of ToxNav on adverse events compared with No-ToxNav***

| Outcome | Odds Ratio | Standard error | P-value | 95%CI | n |
| --- | --- | --- | --- | --- | --- |
| HB_g1_p_rate | 0.91 | 0.09 | 0.311 | 0.76 - 1.09 | 1,604 |
| HB_g2_p_rate | 0.59 | 0.08 | 0.000 | 0.45 - 0.77 | 1,604 |
| HB_g3_p_rate | 0.55 | 0.14 | 0.018 | 0.33 - 0.90 | 1,604 |
| neutrophil_g1_p_rate | 1.01 | 0.13 | 0.964 | 0.77 - 1.31 | 1,604 |
| neutrophil_g2_p_rate | 1.73 | 0.27 | 0.001 | 1.27 - 2.35 | 1,604 |
| neutrophil_g3_p_rate | 1.35 | 0.26 | 0.118 | 0.93 - 1.97 | 1,604 |
| neutrophil_g4_p_rate | 0.77 | 0.25 | 0.419 | 0.40 - 1.46 | 1,604 |
| temperature_g1_p_rate | 0.80 | 0.29 | 0.534 | 0.40 - 1.62 | 1,373 |
| temperature_g2_p_rate | 0.80 | 0.29 | 0.534 | 0.40 - 1.62 | 1,373 |
| temperature_g3_p_rate | 2.97 | 4.12 | 0.432 | 0.20 - 44.94 | 1,373 |
| wcc_g1_p_rate | 0.96 | 0.12 | 0.768 | 0.76 - 1.22 | 1,603 |
| wcc_g2_p_rate | 1.23 | 0.19 | 0.160 | 0.92 - 1.66 | 1,603 |
| wcc_g3_p_rate | 1.10 | 0.25 | 0.690 | 0.70 - 1.72 | 1,603 |
| wcc_g4_p_rate | 0.47 | 0.21 | 0.096 | 0.19 - 1.14 | 1,603 |
| Pain | 0.50 | 0.13 | 0.01 | 0.30 - 0.83 | 979 |
| Sickness | 1.25 | 0.55 | 0.61 | 0.53 - 2.97 | 977 |
| Disturbed sleep | 0.80 | 0.19 | 0.35 | 0.49 - 1.28 | 980 |
| Fatigue | 0.76 | 0.17 | 0.22 | 0.49 - 1.19 | 980 |
| PHQ-8 | 1.11 | 0.23 | 0.62 | 0.74 - 1.65 | 980 |

Note: all regressions include the confounders and are weighted with the Propensity Score

**Appendix I ToxNav operational delivery teams**

**Oxford University Hospitals NHS Trust**

Kristen Moorhouse

Nicola Stoner

Andrew Weaver

Rebecca Muirhead

Clare Jacobs

Rachel Kerr

David Church

Nicola Warner

Kinnari Patel

Robert Owens

Shivan Shivkumar

Somanth Mukherjee

Sileida Oliveros

Rene Roux

Hayley Smith

**Oxford Cancer Biomarkers**

Sam Tritton

Pawel Wolyniec

Kevin Xu

Susan Fotheringham

Guy Mozolowski

1. Kerr RS, Love S, Segelov E, et al. Adjuvant capecitabine plus bevacizumab versus capecitabine alone in patients with colorectal cancer (QUASAR 2): an open-label, randomised phase 3 trial. *Lancet Oncol* 2016;17(11):1543-57. doi: 10.1016/S1470-2045(16)30172-3 [published Online First: 2016/09/24]

2. Rosmarin D, Palles C, Church D, et al. Genetic markers of toxicity from capecitabine and other fluorouracil-based regimens: investigation in the QUASAR2 study, systematic review, and meta-analysis. *J Clin Oncol* 2014;32(10):1031-9. doi: 10.1200/JCO.2013.51.1857 [published Online First: 2014/03/05]

3. Palles C, Fotheringham S, Chegwidden L, et al. An Evaluation of the Diagnostic Accuracy of a Panel of Variants in DPYD and a Single Variant in ENOSF1 for Predicting Common Capecitabine Related Toxicities. *Cancers (Basel)* 2021;13(7) doi: 10.3390/cancers13071497 [published Online First: 2021/04/04]

4. Lee LYW, Starkey T, Sivakumar S, et al. ToxNav germline genetic testing and PROMinet digital mobile application toxicity monitoring: Results of a prospective single-center clinical utility study-PRECISE study. *Cancer Med* 2019;8(14):6305-14. doi: 10.1002/cam4.2529 [published Online First: 2019/09/06]

5. Stuart EA. Matching methods for causal inference: A review and a look forward. *Statistical science : a review journal of the Institute of Mathematical Statistics* 2010;25(1):1-21. doi: 10.1214/09-STS313

6. Garrido MM, Kelley AS, Paris J, et al. Methods for constructing and assessing propensity scores. *Health Serv Res* 2014;49(5):1701-20. doi: 10.1111/1475-6773.12182

7. Baser O. Too much ado about propensity score models? Comparing methods of propensity score matching. *Value Health* 2006;9(6):377-85. doi: 10.1111/j.1524-4733.2006.00130.x

8. Craig P, Cooper C, Gunnell D, et al. Using natural experiments to evaluate population health interventions: new Medical Research Council guidance. *J Epidemiol Community Health* 2012;66(12):1182-6. doi: 10.1136/jech-2011-200375

9. Rubin DB. Using propensity scores to help design observational studies: application to the tobacco litigation. *Health Services & Outcomes Research Methodology* 2001;2:169-88.

10. Funk MJ, Westreich D, Wiesen C, et al. Doubly robust estimation of causal effects. *Am J Epidemiol* 2011;173(7):761-7. doi: 10.1093/aje/kwq439
